# A Case of Pyriform Sinus Fistula with Respiratory Distress in the Neonatal Period

**DOI:** 10.1155/2018/1696875

**Published:** 2018-06-21

**Authors:** Noriko Hamaguchi, Hajime Ishinaga, Kazuki Chiyonobu, Hiroyuki Morishita, Kazuhiko Takeuchi

**Affiliations:** Department of Otorhinolaryngology-Head and Neck Surgery, Mie University Graduate School of Medicine, 2-174 Edobashi, Tsu, Mie 514-8507, Japan

## Abstract

Pyriform sinus fistula (PSF) is an anomaly that can arise due to failure of involution of the third or fourth branchial cleft during embryogenesis. It can manifest clinically as sinuses, cysts, or abscesses in the neck and is common in childhood. Herein, we describe a neonate who presented with neck swelling and respiratory distress, which was secondary to a fourth branchial pouch sinus. Physical examination revealed swollen areas in the posterolateral pharyngeal wall and on the external left side of the neck. Computed tomography imaging showed a left-sided mass that was filled with air and fluid. Eventually, the pyriform sinus cyst and the entire fistulous tract were excised. The postoperative course was uneventful. Follow-up after 18 months showed no recurrence.

## 1. Introduction

Branchial arch anomalies are the second most common congenital abnormality, representing 17% of congenital anomalies in the head and neck region in the pediatric population [1]. Third and fourth arch anomalies are rare, constituting approximately 2% of branchial anomalies, and present as recurrent neck abscesses or suppurative thyroiditis in older children. In neonates, pyriform sinus fistula (PSF) is sometimes referred to as a pyriform sinus cyst, which may cause stridor and respiratory compromise [2]. Herein, we describe a case of PSF in a 9-day-old infant who presented with respiratory distress and underwent an operation on day 30.

## 2. Case Report

**Figure 1:**
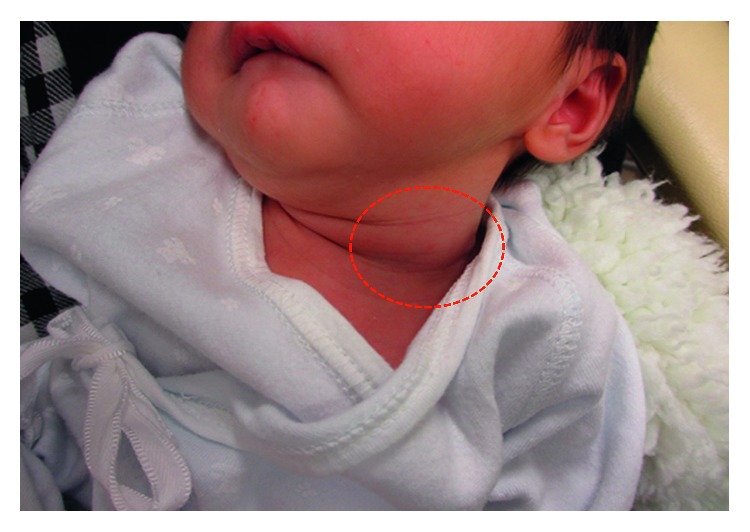
Left cervical swelling (red circle) is seen on physical examination.

**Figure 2:**
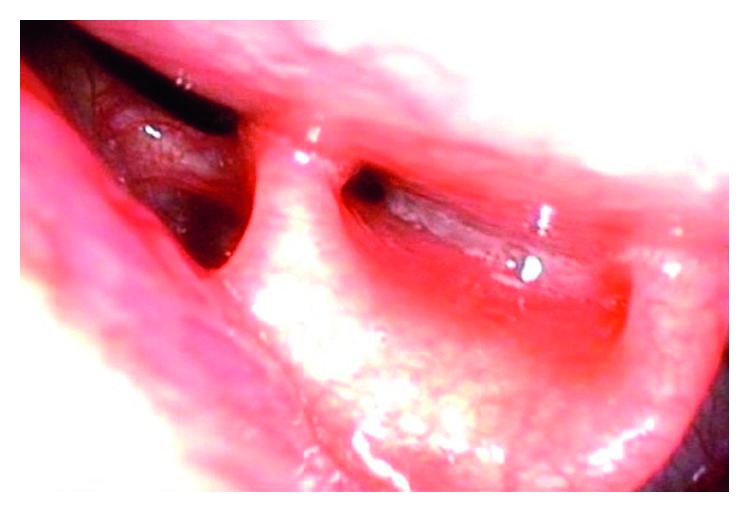
Posterior pharyngeal wall swelling was detected by preoperative laryngoscopy.

**Figure 3:**
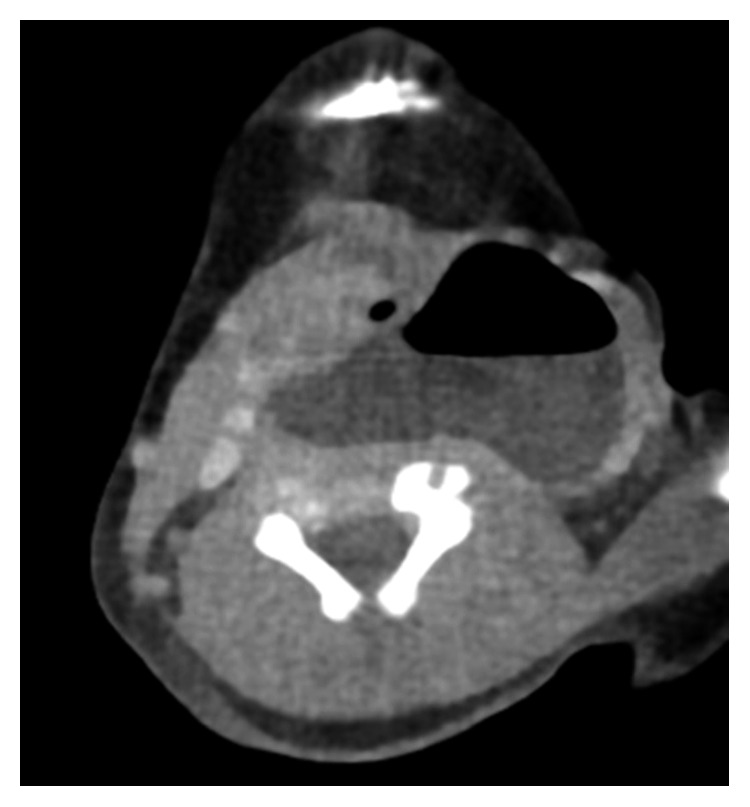
Computed tomography scan showed a cystic lesion on the left side of the neck. An air-fluid level could be detected within the cyst.

The perinatal period of this patient was normal and delivered on 39 week 4 day through the vagina. The Apgar score was full marks. On day 9, the patient developed neck swelling with approximately 5 cm soft mass (Figure 1) and inspiratory stridor and was referred to our department. His body weight was 3024 g, and he had lost 76 g since birth. Arterial saturation was maintained between 95% and 99% in a supine position but decreased to 85% to 90% in a right lateral decubitus position. Physical examination revealed swelling of the posterior pharyngeal wall, which caused extrinsic compression of the airway (Figure 2). There was no paralysis of the recurrent laryngeal nerve. Computed tomography (CT) (Figure 3) and magnetic resonance imaging (MRI) revealed a cystic lesion with air-fluid level, approximately 60 × 50 mm in size, on the left side of the neck, which extended from the epipharynx to the superior mediastinum. Local infection was not suspected both on physical examination and laboratory findings. These findings strongly suggested PSF.

For immediate relief of respiratory distress, puncture of the mass was performed under ultrasound guidance, enabling aspiration of 20 ml of exudate, which resulted in improvement of the respiratory conditions and feeding. Bacteriological examination with the aspirated sample showed no growth in any type of bacteria. However, recurrence of the symptoms a few days later prompted surgical treatment. On day 30, we performed PSF removal via a cervical approach. Though it was difficult to identify the opening of the pyriform fossa under rigid laryngopharyngoscopy, the pyriform sinus cyst and fistula could be visualized intraoperatively by open surgery. The pyriform sinus cyst adhered to the left lobe of the thyroid gland; therefore, both of them were resected. One postoperative complication was transient paralysis of the left recurrent laryngeal nerve, which resolved after 6 days. There was no recurrence of symptoms within 18 months after the operation.

## 3. Discussion

A lateral cervical cyst in a neonate has several differential diagnoses, including PSF, lateral cervical cyst, branchial cyst, bronchogenic cyst, vascular malformation and thymic cyst, foregut duplication cyst, and external laryngocele [1]. Most third or fourth branchial cleft cysts appear on the left side, are enlarged, and contain air bubbles visible on CT or MRI scans, which are useful modalities for the diagnosis of PSF [2]. The other examinations that have been reported to be valuable for identification of the fistula in order to make a definite diagnosis of PSF are barium swallow study or direct laryngoscopy [3]. However, in our case, the latter two examinations were difficult to perform preoperatively because the patient was in respiratory distress. Moreover, in general, cooperation with these examinations is difficult in the neonatal period.

Incision and drainage are often selected for the initial treatment of infected branchial cleft cysts; however, these have been shown to have high recurrence rates [4]. Total excision of the cyst and fistula is the best way to achieve a radical cure, but it should be noted that postoperative complications can sometimes occur after PSF excision. Recently, endoscopic electrocautery or laser cautery has been reported as an excellent treatment for infected PSF in children [4, 5]. A systematic review found that total excision by open surgery had higher rates of postoperative complications in children less than 8 years of age than in older children [3]. Regarding the treatment of PSF in neonates, it still remains controversial whether initial drainage with subsequent surgical removal is better or immediate operation. Mouri et al. described that operation in the neonatal period could be safely performed without complications [6]. They concluded that a more rapid cure can be achieved by early complete surgical intervention. In our case, total excision could be performed without severe postoperative complications, too. Therefore, we concluded that a total excision for PSF in neonates would be an effective and alternative method.
